# Catalogue of Members American Med. Association

**Published:** 1881-03-20

**Authors:** 


### *CATALOGUE OF MEMBERS OF THE AMERICAN MEDICAL ASSOCIATION.*

The following is an analysis of the catalogue of members of the "American Medical Association," by T. B. Greenley, M. D., of Kentucky:

That old and indefatigable student, Dr. Toner, of the U. S. Army, has arranged in alphabetical order, and by States, a catalogue of the members of the American Medical Association, from its formation to 1880, inclusive.

Thinking an analysis of the catalogue might be of interest to the profession, I have spent some time and a great deal of pains in analyzing it in its various phases. In doing so I have given the aggregate number of the members, the number of each State and foreign countries; the number of deaths, both in the aggregate and for each State. Also the number becoming members each year since the commencement.

The whole number of members up to, and including 1880, is 7,294. The number of deaths, 1,314.

The number of members from each State, and also the deaths, are as follows:

| | | | |
|---|---|---|---|
| Alabama, | 97; | deaths, | 19. |
| Arkansas, | 48; | " | 3. |
| California, | 93; | " | 13. |
| Colorado, | 15; | " | 1. |
| Connecticut, | 234; | " | 83. |
| Delaware, | 39; | " | 12. |
| District of Columbia, | 121; | " | 31. |
| Florida, | 8; | " | 00. |
| Georgia, | 125; | " | 19. |
| Illinois, | 448; | " | 31. |
| Indiana, | 345; | " | 15. |
| Iowa, | 1[illegible]8; | " | 12. |
| Kansas, | 19; | " | 1. |
| Kentucky, | 256; | " | 30. |
| Louisiana, | 70; | " | 9. |
| Maine, | 91; | " | 23. |
| Maryland, | 235; | " | 65. |
| Massachusetts | 590; | " | 196. |
| Michigan | 270; | " | 26. |
| Minnesota, | 36; | " | 6. |
| Mississippi | 56; | " | 12. |
| Missouri, | 219; | " | 10. |
| Nebraska, | 6; | " | 00. |
| New Hampshire, | 107; | " | 36. |
| New Jersey, | 209; | " | 47. |
| New York, | 1183; | " | 183. |
| North Carolina, | 41; | " | 2. |

| | | | |
|---|---|---|---|
| Ohio | 470; | " | 90. |
| Oregon, | 6; | " | 1. |
| Pennsylvania, | 773; | " | 140. |
| Rhode Island, | 73; | " | 22. |
| South Carolina, | 92; | " | 28. |
| Tennessee | 151; | " | 34. |
| Texas, | 35; | " | 3. |
| Vermont, | 105; | " | 18. |
| Virginia, | 186; | " | 46. |
| West Virginia, | 34; | " | 5. |
| Wisconsin, | 80; | " | 10. |
| Territories, | 6; | " | 00. |
| U. S. Army | 42; | " | 12. |
| U. S. Navy | 49; | " | 11. |

FOREIGNERS.

| | | | |
|---|---|---|---|
| Central America | 1. | France, | 3. |
| China | 1. | Peru, | 1. |
| Germany, | 1. | Halifax, | 1. |
| Turkey, | 1. | Ireland, | 1. |
| Canada, | 49. | England, | 7. |
| New Brunswick | 2. | Italy, | 1. |
| Brazil, | 1. | Austria, | 1. |

Norway, 1

The number becoming members each year is as follows:

| | | | | | |
|---|---|---|---|---|---|
| 1846 | 126 | 1847 | 189 | 1848 | 189 |
| 1849 | 324 | 1850 | 218 | 1851 | 97 |
| 1852 | 160 | 1853 | 286 | 1854 | 183 |
| 1855 | 229 | 1856 | 214 | 1857 | 125 |
| 1858 | 203 | 1859 | 170 | 1860 | 241 |
| 1863 | 141 | 1864 | 261 | 1865 | 265 |
| 1866 | 121 | 1867 | 211 | 1868 | 132 |
| 1869 | 143 | 1870 | 196 | 1871 | 136 |
| 1872 | 342 | 1873 | 271 | 1874 | 228 |
| 1875 | 236 | 1876 | 307 | 1877 | 297 |
| 1878 | 320 | 1879 | 146 | 1880 | 558 |

By making comparative examination of the number of deaths in each State, it will be observed that a great difference exists as to the per cent. of mortality. This, I think, mainly depends on the inability of the statistician to learn the history of members who joined many years ago. A great many no doubt changed localities, thus rendering it a difficult matter, in many instances, to trace them. The mortality ranges as low as 4½ per cent in Indiana, and as high as 36 per cent. in Connecticut. This is certainly too great a difference to be correct.

It is said by statisticians that the average life of a physician is 56 years, but not having the ages of all who died belonging to the Association, I cannot make a satisfactory estimate.

Dr. Toner, in his report on necrology, gives a short biography of 72 members. I find their ages to have averaged 58.3 years.

It will be noticed that no meetings of the Association were held in 1861 and 1862. These exceptions were due to our unfortunate civil war. It will also be observed that there exists a great difference in the number who joined at the various meetings. This was dependent very much on the locality of the meetings. When the Association assembled at places central to a dense population, then large accessions were obtained, and *vice versa.*

A greater number joined last year than ever before, which I think may be accounted for, to some extent, to the increasing popularity of the Association.

The catalogue and biographies above referred to are published in the transactions of the American Medical Association for 1880.

*Ord, Kentucky, February* 22, 1881.

